# Selective Inhibition of Wild Sunflower Reproduction with Mugwort Aqueous Extract, Tested on Cytosolic Ca^2+^ and Germination of the Pollen Grains

**DOI:** 10.3390/plants10071364

**Published:** 2021-07-03

**Authors:** Alberto Marco Del Pino, Euro Pannacci, Alessandro Di Michele, Elisabetta Bravi, Ombretta Marconi, Francesco Tei, Carlo Alberto Palmerini

**Affiliations:** 1 Department of Agricultural, Food and Environmental Sciences, University of Perugia, Via Borgo XX Giugno 74, 06121 Perugia, Italy; alberto.delpino@unipg.it (A.M.D.P.); e_bravi@yahoo.it (E.B.); ombretta.marconi@unipg.it (O.M.); francesco.tei@unipg.it (F.T.); carlo.palmerini@unipg.it (C.A.P.); 2 Department of Physics and Geology, University of Perugia, Via Pascoli, snc, 06123 Perugia, Italy; alessandro.dimichele@unipg.it

**Keywords:** wild sunflower, imidazolinone-tolerant crops, IMI technology, pollen germination, caffeic acid, ferulic acid, new weed management strategy, outcrossing

## Abstract

Wild sunflower (*Helianthus annuus* L.) is an invasive species widely distributed in several regions of the world, where it shares a large area with domesticated sunflower. The imidazolinone-tolerant sunflower enables the control of problematic weeds (such as *Xanthium* spp., *Brassica* spp., wild sunflower) with imidazolinone herbicides (Clearfield^®^ production system) in cultivated sunflower crops, but could facilitate the gene transfer of herbicide resistance, from cultivated sunflower to wild sunflower, generating hard-to-control weed biotypes or herbicide-resistant populations. The development of new practices that involve the selective inhibition of reproduction structures, such as pollen granules, could be an innovative strategy to minimize outcrossing and the origin of weed–crop hybrids in Clearfield^®^ production systems. In this study, the effects of mugwort (*Artemisia vulgaris* L.) aqueous extract on cytosolic Ca^2+^ and the germination of pollen grains collected from conventional, wild and IMI-tolerant sunflower were tested. The results showed that mugwort deregulated Ca^2+^ homeostasis and markedly reduced the germination of conventional and wild sunflower pollen, but not IMI-tolerant pollen. The HPLC analysis revealed the presence of phenolic acids belonging to the hydroxycinnamic and benzoic classes in the mugwort extract. Hydroxycinnamic acids (caffeic and ferulic) deregulated the cytosolic Ca^2+^ of conventional and wild sunflower pollen, but not those which were IMI-tolerant, similar to mugwort extract. Selective inhibition of wild sunflower pollen in the Clearfield^®^ sunflower crop contributes to a possible new weed management strategy, reducing the wild sunflower reproduction by seed, minimizing the potential risks of outcrossing with the formation of weed–crop hybrids. The Ca^2+^ selective chelating activity of caffeic or ferulic acids provides elements to be investigated for their possible use as an alternative to mugwort extract.

## 1. Introduction

Wild sunflower (*Helianthus annuus* L.) is an invasive species widely distributed in several regions of the world, including several main sunflower-producing countries, such as Argentina, France, Italy, Serbia and Spain, where it shares a large area with domesticated sunflower [1]. Wild sunflower seeds are dormant following dispersal, and up to 40% of the seeds may remain dormant during the spring germination period, surviving from 1 to 10+ years in the soil, so as to form persistent seed banks [2,3]. Wild sunflower control, in conventional sunflower varieties, is very difficult and mainly based on non-chemical strategies, such as false seedbed technique and mechanical methods, because pre- and post-emergence herbicides are not effective [4,5]. In fact, both pre-emergence herbicides applied when sowing sunflower crops to prevent the germination of weed seeds and post-emergence herbicides applied after sunflower emergence to kill weeds after they have germinated are not selective on cultivated sunflowers, and at the same time, are effective against wild sunflower. For these reasons, the false seed-bed technique is used to manage wild sunflower. This technique consists of preparing a regular seedbed early and then—instead of sowing the crop directly—allows the weeds and wild sunflower to germinate and then control them repeatedly before sowing the sunflower crop. After cultivated sunflower emergence, the subsequent emergences of wild sunflower plants can be controlled by inter-row mechanical methods such as hoeing and ridging. The discovery of imidazolinone herbicides resistance in a wild sunflower population enabled the development of Clearfield^®^ technology in cultivated sunflower [6]. The Clearfield^®^ production system, by combining imidazolinone-tolerant crops with imidazolinone herbicides, is able to control certain weeds that no other herbicide can control in some crops. Imazethapyr and imazamox control several problematic broadleaf weeds such as *Xanthium* spp., *Brassica* spp. and wild sunflower in cultivated sunflowers, giving farmers more flexibility to grow sunflowers in areas where broadleaf weeds are problems or where soil-applied herbicides are not compatible with conservation tillage practices [7]. Imidazolinone herbicides control weeds by inhibiting the enzyme acetohydroxyacid synthase (AHAS), also called acetolactate synthase (ALS), which is a critical enzyme for the biosynthesis of branched chain amino acids in plants. Furthermore, imidazolinone-tolerant plants with altered AHAS genes and enzymes have been discovered in many crops. This makes it possible to develop imidazolinone-tolerant crops based on the resistance mechanism at the site of action for these crops [7]. IMI technology was rapidly adopted worldwide, but the trait could possibly be transferred to wild populations through natural hybridization [6]. Genes can be exchanged between plants of the same or sometimes different species through crosspollination. As a result, outcrossing of certain crop traits such as herbicide resistance to closely related weed plants or gene flow from crops to weed species is a major concern [7]. Given the existence of wild *H. annuus* populations established in several regions of the world, and the possibility of breeding fertile hybrids with cultivated sunflowers, the widespread usage of IMI technology could favor the gene transfer of herbicide resistance from crops to wild species, generating noxious weed biotypes difficult to control [6]. Concerns about gene flow from cultivated sunflower to wild sunflower are particularly relevant to imidazolinone-tolerant crops. If weeds gain the herbicide-tolerance trait from the crops, the herbicide will fail to control the weeds effectively and may result in herbicide-resistant populations. Therefore, herbicide-resistant crop systems must be integrated with proper stewardship to minimize outcrossing and the survival of weed–crop hybrids, ensuring long term success of the system [7]. Stewardship for the Clearfield^®^ production system may include both required and recommended practices, such as strict guidelines for seed producers of imidazolinone-tolerant crops, rotating crops and rotating herbicides for growers, as well as controlling key weeds and imidazolinone-tolerant volunteer plants in areas adjacent to imidazolinone-tolerant crops [7].

In this context, the development of new practices that involve the selective inhibition of reproduction structures, such as pollen granules, could be an innovative strategy to minimize outcrossing and the origin of weed–crop hybrids in Clearfield production systems.

Male microgametophytes (i.e., pollen grain) are a specialized sexual system based on the transport and remote delivery of large quantities of pollen. This reproduction system allows habitat colonization and adaptation to dry environments [8]. Pollen grains are driven by many signals to the haploid cell (the female gametophyte), and cytosolic Ca^2+^ is of paramount importance in signal–response coupling [8,9].

Numerous studies have described the physiological role of cytosolic Ca^2+^ as a secondary messenger in the growth and formation of pollen tubes [8,9,10,11,12]. Transient increases in cytosolic Ca^2+^ activate cellular responses [10,13]; therefore, levels of cytosolic Ca^2+^, under non-stimulus conditions, must be maintained at concentrations below 0.1 μM. In the plasma membrane of the plant cell, several channels regulate Ca^2+^ entry; however, despite the permeability of the ion, none of these channels appear to be Ca^2+^-specific [14]. Ca^2+^ enters the cell through these channels and activates vertical cell growth [8]. The correlation between cytosolic Ca^2+^ levels and pollen germination is widely described in the literature [8,10,11,15,16,17], and the formation of a Ca^2+^ gradient activates pollen germination [18,19].

The study of the effects of mugwort (*Artemisia vulgaris* L.) presented in this work was possible due to the knowledge gained in previous studies on mugwort activity against weeds [20,21].

Mugwort is a perennial herb that grows spontaneously and abundantly in the temperate and cold areas of the world. It is an edible plant used in medicine as an anti-allergic, anti-inflammatory and antimicrobial agent. Mugwort also produces volatile allelochemicals with a negative impact on surrounding vegetation by inhibiting the growth and survival of other plants [22,23].

Some pilot studies conducted in our laboratory have shown, for the first time, an effect of mugwort on cytosolic Ca^2+^ in pollen. Therefore, the mugwort aqueous extract was tested on cytosolic Ca^2+^ and on the germination of wild, conventional and IMI-tolerant sunflower pollen grains, with the aim of verifying the existence of a selective biological activity to be employed in new practices in the management strategies of wild sunflower.

## 2. Results

### 2.1. Morphology of Sunflower Pollen Grains

**Figure 1:**
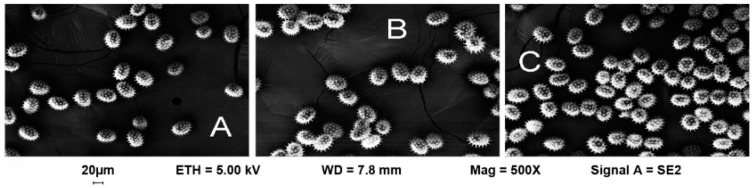
The morphology of pollen grains showed by scanning electron microscopy (500× magnification) in conventional (**A**), wild (**B**) and IMI-tolerant (**C**) sunflower populations.

The morphological analysis of conventional, wild and IMI-tolerant sunflower pollen was investigated by field emission scanning electron microscopy. The images (500× magnification) showed that the size and shape of the pollen grains were typical of sunflower pollen, and that there are no macroscopic differences between the three different populations of sunflower (Figure 1).

### 2.2. Effect of Mugwort on Cytosolic Ca^2+^ ([Ca^2+^]cp) in Sunflower Pollen Grain

The pollen grains of the different types of sunflower (conventional, wild and IMI-tolerant) were marked with the FURA-2AM fluorescent probe to determine the variation of the cytosolic Ca^2+^ ([Ca^2+^]cp).

The determination of [Ca^2+^]cp was carried out in two phases, in the absence and presence of Ca^2+^ in the incubation medium with resuspended aliquots of pollen. Aliquots of mugwort (0.4, 0.78, 1.56, 3.12, 6.25, 12.5 mg) were added “in vitro” in the essay to the suspension of conventional, wild and IMI-tolerant sunflower pollen.

The pollen suspension, labeled with FURA 2AM, was placed in a cuvette and the fluorescence was measured until the signal stabilization was reached (50 s). After the addition of the mugwort extract, the fluorescence was measured for another 150 s, at the end of which the signal had returned to baseline levels (see the Section 4).

**Figure 2:**
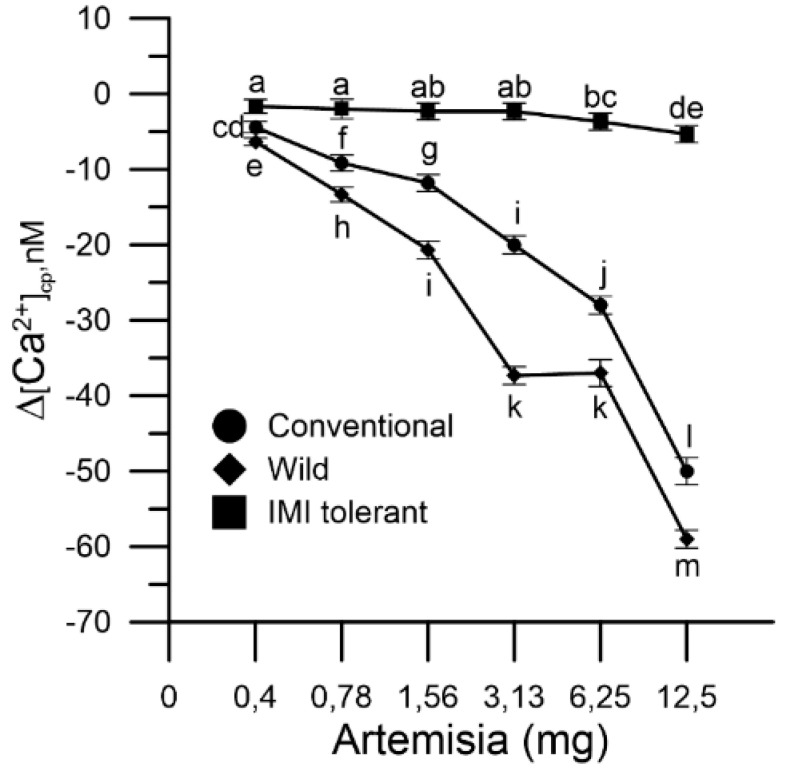
Effect of mugwort on levels of cytosolic Ca^2+^ in sunflower pollen grains: conventional (●), wild (♦) and ‘IMI-tolerant’ (■). Results are expressed as ∆[Ca^2+^]cp (nM) to reflect signal changes detected between the start and the end of the fluorometric measurements and represent means ± SEM from three independent tests. Statistically significant differences are indicated by different letters, whereas identical letters highlight non-significant trends. SEM, standard error of the mean.

Basal cytosolic Ca^2+^ levels of wild and conventional sunflower pollen decreased, in a dose-dependent manner, in the presence of mugwort extracts in the incubation medium, whereas IMI-tolerant pollen was not affected (Figure 2). The Ca^2+^ chelating effect of mugwort extracts in the three sunflower pollen populations was in the order: wild > conventional > IMI-tolerant. It should be emphasized that the variations produced in the cytosolic Ca^2+^ of IMI-tolerant sunflower pollen were modest and appreciable only at high concentrations of mugwort (6.25 and 12.5 mg) (Figure 2).

**Figure 3:**
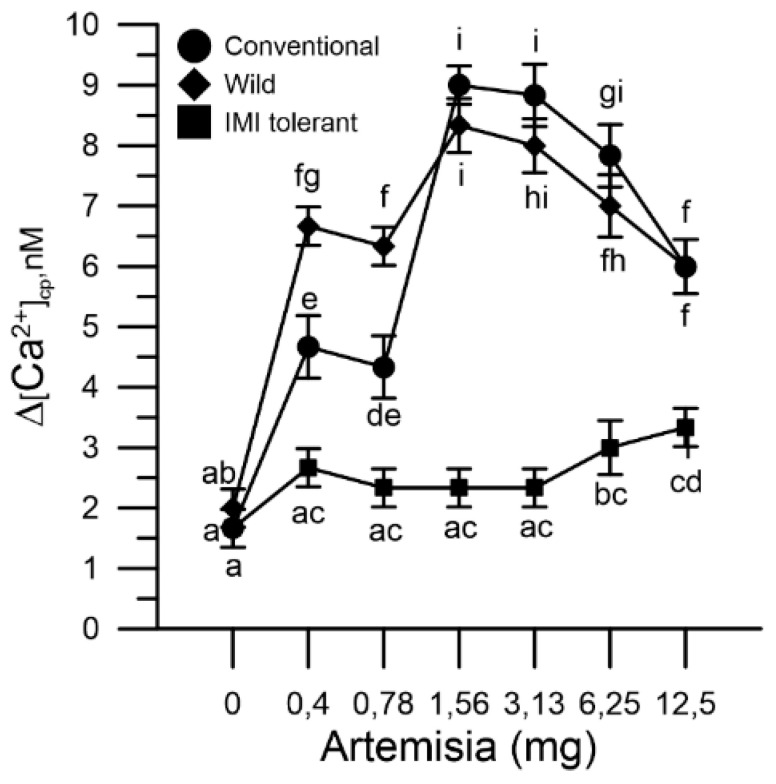
Effect of mugwort in the Ca^2+^ entry of different sunflower pollen grain: conventional (●), wild (♦) and ‘IMI-tolerant’ (■), after the addition of CaCl_2_ to the incubation medium. The results are expressed as ∆[Ca^2+^]cp (nM), reporting the signal changes detected between the beginning and the end of the fluorometric measurements (1 min) and represent the means ± SEM of three independent tests. Statistically significant differences are indicated by different letters, whereas identical letters highlight non-significant trends. SEM, standard error of the mean.

In the same determination, at the end of the mugwort effect, CaCl_2_ 1 mM was added. The increase in cytosolic Ca^2+^ produced by the entry of the ion from the extracellular medium (Ca^2+^ entry) was monitored until the signal stabilized (250 s) [24]. The Ca^2+^ entry was influenced by the extent of the Ca^2+^ chelating effect produced by mugwort. Internal reserves of conventional and wild sunflower pollen, more depleted by mugwort, showed higher Ca^2+^ entry than those of IMI-tolerant sunflower pollen (Figure 3).

### 2.3. Content in Phenolic Acids of the Mugwort Extract

**Figure 4:**
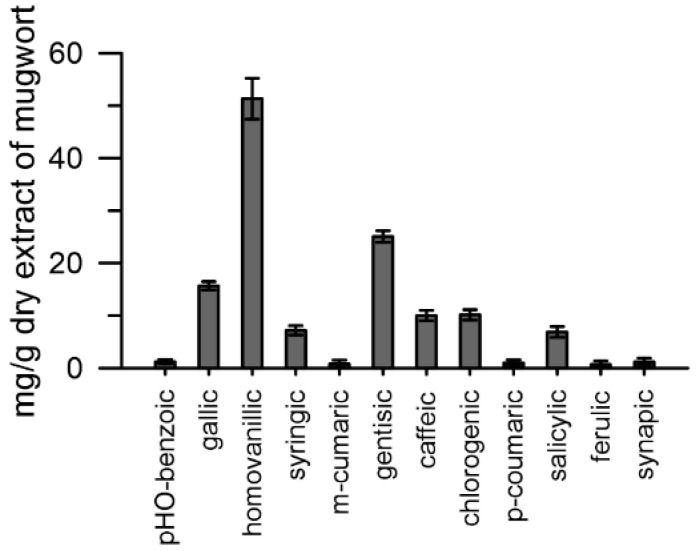
Phenolic acids present in the aqueous extract of mugwort. Data are expressed as means ± SEM from three independent tests.

Aliquots of the mugwort extract (25%) were injected into a UHPLC system (see Section 4). The results obtained highlighted twelve different phenolic compounds in the mugwort extract: hydroxycinnamic acids (caffeic, synapic, p/m -coumaric, ferulic, homovanillic, and chlorogenic) and hydroxybenzoic acids (p-hydroxybenzoic, gallic, syringic, salicylic, and gentisic) (Figure 4).

The phenolic acids present in the highest quantities were: homovanillic > gentisic > gallic. Caffeic, chlorogenic, salicylic and syringic were present in amounts between 8 and 10 mg·g^−1^ dry tissue, whereas p/m-coumaric, ferulic, synaptic and p-hydroxybenzoic were the least abundant, between 2 and 3 mg·g^−1^ dry tissue (Figure 4).

### 2.4. Effects of Hydroxy Cinnamic Acids in the Cytosolic Ca^2+^ of Sunflower Pollen

On the basis of previous investigations [25], the caffeic and ferulic standards (5 mg mL^−1^) were chosen, among the hydroxycinnamic acids present in mugwort, to verify the chelating effects on the cytosolic Ca^2+^ of the three sunflower pollen populations.

The same experimental protocol used to determine the effects of mugwort on the cytosolic Ca^2+^ of pollen was used to test the phenolic acid purity. After stabilizing the signal of the pollen suspension labeled with the FURA 2AM for 50 s, each pure hydroxycinnamic acid was added to the cuvette and the effects on the cytosolic Ca^2+^ were measured until the return to basal values (150 s) (see Section 4).

**Figure 5:**
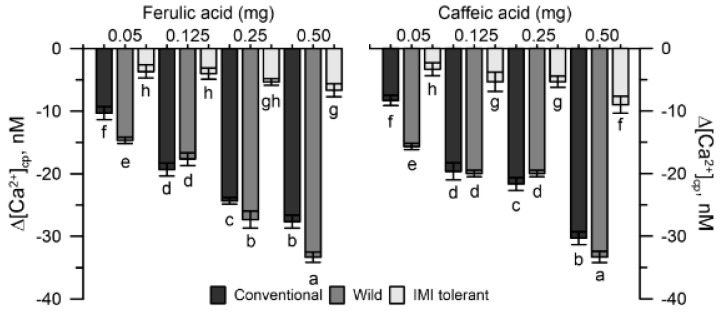
Effects of ferulic acid and caffeic acid on the cytosolic Ca^2+^ of conventional, wild and IMI-tolerant sunflower pollen. Data are expressed as means ± SEM from 4 independent tests. Statistically significant differences are indicated by different letters, whereas identical letters highlight non-significant trends. SEM, standard error of the mean.

Pollen from conventional and wild sunflowers was more sensitive to the Ca^2+^ chelating activity of ferulic or caffeic acid, whereas pollen from IMI-tolerant sunflowers was much less sensitive (Figure 5).

### 2.5. Germination of Sunflower Pollen

**Figure 6:**
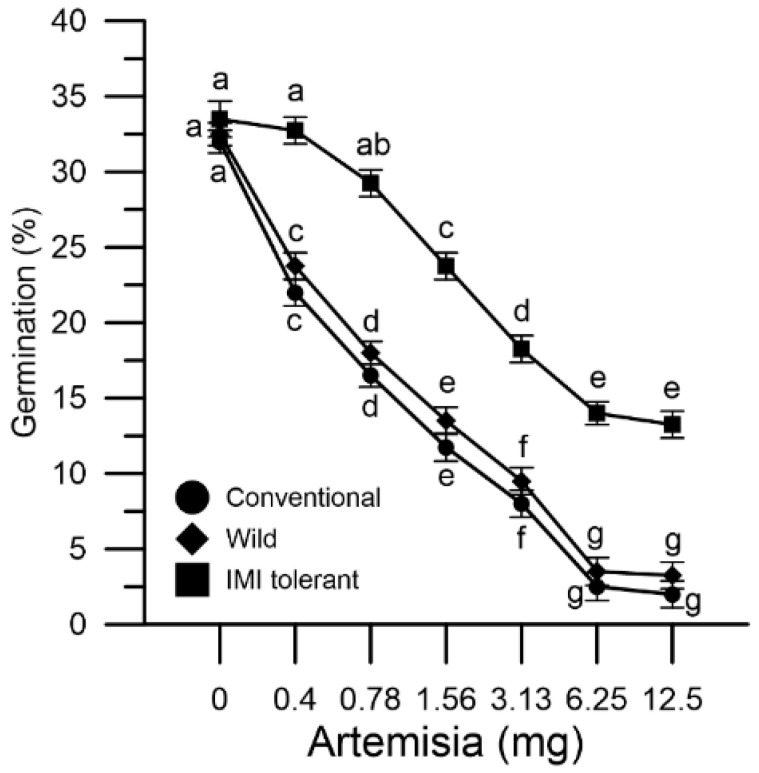
Effect of mugwort on the germination of different sunflower pollen grains: conventional (●), wild (♦) and ‘IMI-tolerant’ (■). Results are expressed in percentages and represent means ± SEM from four independent tests. Statistically significant differences are indicated by different letters, whereas identical letters highlight non-significant trends. SEM, standard error of the mean.

Pollen grains collected from conventional, wild and IMI-tolerant sunflowers were incubated in vitro with mugwort extracts (from 0.4 to 12.5 mg). Mugwort influenced pollen germination by decreasing it in a dose-dependent manner. The extent of the effect in the three pollen populations was different (Figure 6).

The germination rate was reduced in vitro by 34% in wild sunflower pollen and by 37% in conventional sunflower pollen, whereas it was reduced by 6% in the IMI-tolerant sunflower with 0.4 mg mugwort. As the mugwort concentration increased, the marked effect on germination in the two mugwort-sensitive populations compared to the IMI-tolerant sunflower persisted (Figure 6).

## 3. Discussion

The wild sunflower (*H. annuus*) is an invasive species whose control, in convention-al sunflower varieties, is very difficult. The development of Clearfield^®^ technology has enabled weed control in crops, but does not exclude the risk of exporting resistance by gene transfer [6]. The development of new practices involving the selective inhibition of reproductive structures could reveal new innovative strategies in controlling pollen grains. The relationship between cytosolic Ca^2+^ and germination in pollen is widely described in the literature [18,19] and can provide useful information on wild sunflower control.

In this work, cytosolic Ca^2+^ and the germination of pollen from conventional, wild and IMI-tolerant sunflowers was tested with mugwort.

The extracts caused a dose-dependent decrease in cytosolic Ca^2+^ ([Ca^2+^]_cp_) in wild and conventional sunflower pollen only.

Mugwort thus deregulated the Ca^2+^ homeostasis of conventional and wild pollen, but not that of IMI-tolerant pollen. Work on the calcium-chelating properties of mugwort and on the molecular effects in the three sunflower pollen populations are not present in the literature. The results reported here suggest a possible different selectivity of the pollen membranes in internalizing the extracts of mugwort and pure hydroxycinnamic acids (ferulic and caffeic), to which the different biological responses can be attributed. The experimental protocol adopted then highlighted that the perturbation of the cytosolic Ca^2+^, in the two mugwort-sensitive sunflower populations determines the exhaustion of the pollen stores of Ca^2+^. This is compensated by an increased entry of Ca^2+^ from the extracellular medium.

The mugwort acted, in a selective way, both on the cytosolic Ca^2+^ and on the germination of the three pollen populations. In fact, an altered Ca^2+^ homeostasis was followed by a marked inhibitory effect on pollen germination (wild and conventional sunflowers) and less altered Ca^2+^ homeostasis was followed by the modest inhibition of germination (IMI-tolerant sunflower).

The link between cytosolic Ca^2+^ and germination of the three sunflower pollen populations was in agreement with what was reported in our previous studies and in those of other authors [18,19,26,27].

Weed control through the selective inhibition of reproductive structures such as sunflower pollen appears to be a promising and easy-to-execute strategy. The possibility of using aqueous mugwort extract in the treatment of wild sunflower plants during flowering would result in the production of pollen with poor germinability, thus reducing the risk of gene flow between wild and IMI-tolerant sunflowers.

However, before implementing the field use of artemisia extracts, toxicity tests and further studies on the mechanism of selectivity shown with sunflower populations will be necessary.

The next phase of this study aimed to analytically identify the possibly active compounds of mugwort.

The chromatographic analysis by HPLC of the mugwort extract showed the presence of phenolic acids belonging to the class of hydroxycinnamic and benzoic acids.

The hydroxycinnamic acids (ferulic and caffeic standards) and the benzoic acids (salicylic and pHO-benzoic standards) tested on the cytosolic Ca^2+^ of sunflower pollen were differently active. Caffeic and ferulic acid reduced cytosolic Ca^2+^ levels, whereas salicylic and pHO-benzoic increased cytosolic Ca^2+^ (data not shown).

The Ca^2+^ chelating activity of caffeic and ferulic was higher in conventional and wild pollen and moderate in IMI-tolerant pollen, similar to that observed with mugwort extract. It is therefore plausible that the Ca^2+^ chelating activity of mugwort can be traced back to the hydroxycinnamic acids present in the extract, as a prevalent species in the unconjugated form [25].

## 4. Materials and Methods

### 4.1. Reagents

FURA 2-AM (FURA-2-pentakis (acetoxymethyl) ester), PBS (Phosphate Buffered Saline), Triton X-100, EGTA (ethylene glycol-bis (β-aminoethyl ether), sodium, hydrogen peroxide (H2O2), sodium chloride (NaCl), potassium chloride (KCl), magnesium chloride (MgCl2), glucose, Hepes, and dimethyl sulfoxide (DMSO) were acquired from the Sigma-Aldrich corporation (St. Louis, MO, USA). Any other chemicals and reagents (reagent grade) were of the highest quality, and obtained from reputable, commercial sources.

### 4.2. Site Description, Treatments and Experimental Design

Sunflowers (*Helianthus annuus* L.) were grown in 2018 in a plant growth chamber at the laboratory of the Agronomy and Crop Sciences Research Unit (Department of Agricultural, Food and Environmental Sciences, University of Perugia), in Perugia (Italy). Three sunflower types were used: imidazolinone-tolerant type (Hybrid SY Experto, Clearfield^®^, Syngenta); conventional type (Hybrid P64LL115, Pioneer); and wild sunflower type. Seeds of wild sunflower were harvested from spontaneous mature plants at the Field-Lab of the University of Perugia located in Papiano (43° N–12° E, elevation 541 ft.).

Quartz sand (11 kg) (inert substrate, 0.2–2 mm mesh size, 1.24 g mL^−1^ bulk density and 38.5 mL 100 mL^−1^ maximum water holding capacity) was used as the substrate to fill each plastic pot (11 L), which then was situated over its saucer. Four seeds of each sunflower type were sown in each pot, in March 2018. These pots were placed in a growth chamber, according to a completely randomized design with three replicates and the following conditions: 20 °C; 75% relative humidity; 11:13 h dark:light regime; light intensity = 200 µmol m^−2^ s^−1^. Pots were sub-irrigated (water delivered below the soil surface to the plant root zone) to the maximum water-holding capacity with distilled water, using saucers as the “subirrigation system”. After emergence, seedlings were thinned to one plant per pot and the water content was daily adjusted to the maximum water-holding capacity by subirrigation with a nutrient solution containing all necessary macro- and micro-elements (Flory 9^®^, Agrimport; 1 g c.f. L^−1^ + urea 0.1 g c.f. L^−1^ + Sequestrene^®^ NK 138 Fe, Ciba-Geigy 0.04 g c.f. L^−1^).

### 4.3. Sunflower Pollen Collection

The pollen grains of sunflowers were collected in June 2018, with the sunflower types at the growth stage 65 of the BBCH scale (full flowering: disc florets in middle third of inflorescence in bloom, stamens and stigmata visible). The collection was carried out by removing the pollen from the sunflower heads with a brush and collecting it on tinfoil. Within ten minutes from collection, pollen from each sunflower type was placed in polypropylene tubes, wrapped with aluminum foil, and immediately transferred into a refrigerated container (5 °C).

### 4.4. Images of Sunflower Pollen Grains by Electron Scanning Microscopy

The morphology of the pollen samples was examined at 500X magnification by field-emission scanning electron microscopy, using a LEO 1525 Gemini workstation (ZEISS) upon metallization with chromium.

### 4.5. Preparation of Mugwort Extract

Plants of mugwort were collected at the growth stage 61–62 of the BBCH scale (beginning of flowering: 10–20% of flowers open) from an uncultivated field in central Italy (42°56′ N, 12°23’ E, 165 m a.s.l.). Fresh mugwort plants were dried in a hot-air oven at 45 °C for 5 days, and aerial biomass (leaves + stems) was ground with an electrical grinder, sieved through a 1 mm sieve, and kept in a dry and dark bag at 10 °C for future use. In the laboratory, aerial biomass at 75 g dry tissue was soaked in 300 mL of distilled water (25% *w*/*v*) for 24 h at 24 °C. After soaking, the aqueous solution was filtered through 4 layers of cheesecloth to remove the fiber debris, and then the aqueous extract was filtered again through filter paper.

The filtrate (stock extract) was diluted with distilled water to obtain the following solutions: 0.8%,1.56%, 3.13%, 6.25%, 12.5% and 25% *w*/*v* concentrations. Aliquots of each solution (corresponding to 0.4, 0.78, 1.56, 3.12, 6.25, 12.5 mg of mugwort dry tissue) were used in the cytosolic Ca^2+^ test carried out on sunflower pollen.

### 4.6. Measurement of Cytosolic Ca^2+^

Intracellular calcium levels were determined spectrofluorometrically using the probe FURA-2AM. Sunflower pollen (100 mg) was suspended in 10 mL PBS and hydrated for 2 days. Hydrated pollen samples were harvested by centrifugation at 1000× *g* for 4 min and then resuspended in 2 mL Ca^2+^-free HBSS buffer (120 mM NaCl, 5.0 mM KCl, MgCl_2_ 1mM, 5 mM glucose, 25 mM Hepes, pH 7.4). Pollen suspensions were incubated in the dark with FURA-2AM (2 µL of a 2 mM solution in DMSO) for 120 min, after which samples were centrifuged at 1000× *g* for 4 min. Pollen was then harvested and suspended in ~10 mL of Ca^2+^-free HBSS containing 0.1 mM EGTA, which was included to rule out, or at least minimize, a potential background due to contaminating ions (so as to obtain a suspension of 1 × 10^6^ pollen granules hydrated per milliliter).

Fluorescence was measured in a Perkin-Elmer LS 50 B spectrofluorometer (ex. 340 and 380 nm, em. 510 nm), set with 10 nm and a 7.5 nm slit widths in the excitation and emission windows, respectively.

The pollen suspension (10^6^) labeled with the FURA 2AM probe was placed in the cuvette under magnetic stirring and the fluorescence was monitored for 50 s. To stabilize the signal, artemisia extracts or pure phenolic acids were added, and the variations of cytosolic Ca^2+^ in pollen were monitored over time. The end of the effect of the agents was evidenced by the return of the cytosolic Ca^2+^ to basal levels after 150 s, in Ca^2+^-free conditions. Subsequently, CaCl_2_ was added, and the rise in cytosolic Ca^2+^ (Ca^2+^ entry) was measured until the signal stabilized after 200 s. At the end of the measurements, the pollen was lysed with Triton X-100 and the total fluorescence was measured (200 s); then, EGTA was introduced and the minimum fluorescence was measured (200 s). The Grynkiewicz equation [28] enabled derivation, from the fluorescence measurements, of the fluctuations of the cytosolic Ca^2+^ ([Ca^2+^]_cp_) (nM) produced by mugwort or pure phenolic acids. The whole measurement carried out continuously and with the same pollen suspension had a duration of 800–1000 s.

### 4.7. Pollen Germination Studies

Fresh pollen samples from each pot were hydrated in a humid chamber at room temperature for 30 min, and then transferred to 6-well culture Corning plates (1 mg of pollen per plate) containing 3 mL of a modified nutrition medium, as suggested by Murthy et al. [29], composed of 1 M sucrose, 3 mM boric acid (H_3_BO_3_), 2 mM potassium nitrate (KNO_3_), 1.5 mM magnesium sulfate (MgSO_4_) and 1 mM calcium chloride (CaCl_2_). The nutrition medium was brought to pH 5.5. Mugwort extracts (0.4, 0.78, 1.56, 3.12, 6.25, 12.5 mg) were added immediately afterwards.

Pollen suspensions were incubated for 48 h in a growth chamber at 25 °C with gentle shaking to ensure homogeneous distribution of the samples in the wells. From 24 h to 48 h, the pollen germination was continuously monitored.

Germinated and non-germinated pollen grains were counted under a 10X magnification microscope. Germination rates were calculated based on four replicates, each of which consisted of one hundred grains. Germination of grains was confirmed when the pollen tube had grown longer than the grain’s diameter [30].

### 4.8. Phenolic Acids (PAs) Determination on Mugwort Extract

Phenolic acids were researched in mugwort extract using the method developed by Bravi et al. [31]. A UHPLC system was utilized, consisting of a Knauer 3950 autosampler with a 10 μL loop, a quaternary Azura P 6.1 L pump (Knauer, Berlin, Germany) coupled with an Azura MWD 2.1 L height channel UV–VIS detector. The separation was carried out using a SunShell C18 column (ChromaNik Technologies Inc., 50 mm × 2.1 mm ID) at 25 °C and a flow rate of 0.4 mL min^−1^. Mobile phase A was 0.1 M citric acid and 0.2 M sodium hydrogen phosphate (85/15; *v*/*v*), and mobile phase B was phase A, methanol and acetonitrile (30/20/50, *v*/*v*/*v*). The pH of mobile phase A was 2.88, and the pH of mobile phase B was adjusted to 3.44 with 85% o-phosphoric acid. The wavelengths of the three channels used for the detection were 254, 278, and 324 nm. Clarity Chromatography Software for Windows (DataApex, Prague, Czech Republic) was used for data acquisition and elaboration. The chromatographic separation was achieved using the following elution gradient: mobile phase A 90% (min 0), 100% (min 2), 70% (min 8), 50% (min 10), 20% (min 12), 90% (min 12.5). The external standard method was used for calibration, and the calibration plots were constructed for standard compounds with a linearity between 0.5 and 5 µg mL^−1^. A stock solution of 100 µg/mL of a mix of considered phenolic acids in methanol/eluent A (30:70, *v*:*v*) was used to prepare working solutions.

### 4.9. Statistical Analysis

All statistical analyses of data were performed using Graph Pad Prism 6.03 software for Windows (La Jolla, CA, USA). Tests for variance assumptions were conducted (homogeneity of variance by Levene’s test, normal distribution by the D’Agostino–Pearson omnibus normality test). Results obtained are expressed as mean values ± standard error of the mean (SEM). Significant differences were analyzed by Fisher’s least significant differences test, after the analysis of variance according to the randomized complete factorial design. Differences with *p* < 0.05 were considered statistically significant.

## 5. Conclusions

The control of weeds through the selective inhibition of reproductive structures such as sunflower pollen is a promising and easy-to-execute strategy. Mugwort’s selective effects on cytosolic Ca^2+^ and the germination of wild sunflower pollen can help minimize the risk of outcrossing and the development of weed–crop hybrids in the Clearfield production system.

Future studies will be able to verify whether the selectivity limited to the examined cultivar can be extended to other Clearfield sunflower cultivars. Before the possible use of mugwort extract in field treatments, it will be necessary to carry out toxicity tests.

The hydroxycinnamic acids with Ca^2+^ chelating activity (caffeic or ferulic) present in the mugwort extract suggest their possible selective use in the treatment of wild sunflower as an alternative to mugwort extract. However, further studies will be needed before using caffeic or ferulic acids in field treatments against wild sunflower during flowering.

## Data Availability

Data sharing is not applicable to this article.
